# Comparison Between Diffusion‐Weighted MRI and ^123^I‐mIBG Uptake in Primary High‐Risk Neuroblastoma

**DOI:** 10.1002/jmri.27458

**Published:** 2020-12-06

**Authors:** Laura Privitera, Patrick W. Hales, Layla Musleh, Elizabeth Morris, Natalie Sizer, Giuseppe Barone, Paul Humphries, Kate Cross, Lorenzo Biassoni, Stefano Giuliani

**Affiliations:** ^1^ Department of Specialist Neonatal and Paediatric Surgery Great Ormond Street Hospital for Children London UK; ^2^ Developmental Imaging and Biophysics Section University College London Great Ormond Street Insitute of Child Health London UK; ^3^ Department of Radiology Great Ormond Street Hospital for Children London UK; ^4^ Nuclear Medicine Physics Clinical Physics, Barts Health NHS Trust London UK; ^5^ Department of Haematology and Oncology Great Ormond Street Hospital for Children London UK

**Keywords:** high‐risk neuroblastoma, ^123^I‐mIBG uptake, apparent diffusion coefficient, diffusion weighted‐imaging, histopathology

## Abstract

**Background:**

High‐risk neuroblastoma (HR‐NB) has a variable response to preoperative chemotherapy. It is not possible to differentiate viable vs. nonviable residual tumor before surgery.

**Purpose:**

To explore the association between apparent diffusion coefficient (ADC) values from diffusion‐weighted magnetic resonance imaging (DW‐MRI), ^123^I‐meta‐iodobenzyl‐guanidine (^123^I‐mIBG) uptake, and histology before and after chemotherapy.

**Study Type:**

Retrospective.

**Subjects:**

Forty patients with HR‐NB.

**Field Strength/Sequence:**

1.5T axial DW‐MRI (b = 0,1000 s/mm^2^) and T_2_‐weighted sequences. ^123^I‐mIBG scintigraphy planar imaging (all patients), with additional ^123^I‐mIBG single‐photon emission computed tomography / computerized tomography (SPECT/CT) imaging (15 patients).

**Assessment:**

ADC maps and ^123^I‐mIBG SPECT/CT images were coregistered to the T_2_‐weighted images. ^123^I‐mIBG uptake was normalized with a tumor‐to‐liver count ratio (TLCR). Regions of interest (ROIs) for primary tumor volume and different intratumor subregions were drawn. The lower quartile ADC value (ADC_25prc_) was used over the entire tumor volume and the overall level of ^123^I‐mIBG uptake was graded into avidity groups.

**Statistical Tests:**

Analysis of variance (ANOVA) and linear regression were used to compare ADC and MIBG values before and after treatment. Threshold values to classify tumors as viable/necrotic were obtained using ROC analysis of ADC and TLCR values.

**Results:**

No significant difference in whole‐tumor ADC_25prc_ values were found between different ^123^I‐mIBG avidity groups pre‐ (*P* = 0.31) or postchemotherapy (*P* = 0.35). In the “intratumor” analysis, 5/15 patients (prechemotherapy) and 0/14 patients (postchemotherapy) showed a significant correlation between ADC and TLCR values (*P* < 0.05). Increased tumor shrinkage was associated with lower pretreatment tumor ADC_25prc_ values (*P* < 0.001); no association was found with pretreatment ^123^I‐mIBG avidity (*P* = 0.17). Completely nonviable tumors had significantly lower postchemotherapy ADC_25prc_ values than tumors with >10% viable tumor (*P* < 0.05). Both pre‐ and posttreatment TLCR values were significantly higher in patients with >50% viable tumor than those with 10–50% viable tumor (*P* < 0.05).

**Data Conclusion:**

^123^I‐mIBG avidity and ADC values are complementary noninvasive biomarkers of therapeutic response in HR‐NB.

**Level of Evidence:**

4.

**Technical Efficacy Stage:**

3.

NEUROBLASTOMA (NB) is the most common extracranial malignant solid tumor of childhood and derives from an aberrant differentiation of the primordial neural crest cells that forms the sympathetic nervous system.^1^ NB represents 8–10% of all childhood tumors and accounts for ~15% of all cancer‐related deaths.^2^ High‐risk neuroblastoma (HR‐NB) is defined as any neuroblastoma (other than stage L1) harboring MYCN amplification or any patient older than 18 months of age at diagnosis with stage M disease.^3^ Due to the significant involvement and encasement of vital organs and vessels by tumor, surgical resection is often high risk and without clear resection margins.^4^, ^5^ It is difficult to predict how much viable tumor is present at the time of surgical resection. Previous studies have demonstrated that tumor shrinkage following chemotherapy may be an inaccurate marker of response, since the number of viable tumor cells is highly variable, with shrinkage not necessarily associated with favorable tumor response.^6^, ^7^, ^8^ This is a challenge, given that large portions of resected tumors are commonly completely necrotic or calcified with no remaining viable tissue, and these areas could be left in place if accurately identified as such with preoperative imaging.^3^, ^9^

^123^I‐meta‐iodobenzylguanidine (^123^I‐mIBG) scintigraphy is widely used in the assessment of neuroblastoma. It has high sensitivity (range 83–92%) and specificity (range 88–92%) at staging.^10^ Moreover, the bulk skeletal disease, assessed with ^123^I‐mIBG using either the International Society of Paediatric Oncology European Neuroblastoma Group (SIOPEN) or the Curie scoring methods, has a prognostic value both at diagnosis and at the end of induction chemotherapy.^11^, ^12^, ^13^ It has also been suggested that ^123^I‐mIBG weakly correlates with tumor differentiation and that ^123^I‐mIBG non‐avid neuroblastoma may have a better prognosis than avid tumors.^14^, ^15^

In addition to nuclear medicine techniques, diffusion‐weighted magnetic resonance imaging (DW‐MRI) can be used in NB to assess the biological features of the tumor. It may be a valuable marker of response after induction chemotherapy.^7^ Initial evidence suggests that tumors with restricted diffusion are likely to behave more aggressively and that less restricted diffusion, with an increased apparent diffusion coefficient (ADC) after induction chemotherapy, is likely to indicate a favorable treatment response.^16^

Currently, the relationship between the ^123^I‐mIBG and DW‐MR imaging characteristics of NB is not known, and it is not possible to confidently use either modality to differentiate between viable and nonviable tumor remaining at the site of primary neuroblastoma after induction chemotherapy.^7^, ^17^ A preoperative imaging technique capable of identifying areas of residual nonviable tumor could potentially minimize unnecessary surgical resections, thus reducing intraoperative morbidity and the length of the operation.

This study aimed to explore the relationships between ADC values, ^123^I‐mIBG uptake, and histopathology in primary HR‐NB before and after chemotherapy.

## Materials and Methods

### Patients

This retrospective study was approved by the Audit Committee of our institution as a service evaluation, waiving the need for informed consent (IRB#2734). Patients with HR‐NB, who received treatment in our hospital between January 2006 and August 2019, were evaluated. The following inclusion criteria were used: pathologically proven HR‐NB at diagnosis with ^123^I‐mIBG scintigraphy and DW‐MRI acquired both at diagnosis and after induction chemotherapy. All patients were staged radiologically at diagnosis with an MRI scan of the body segment where the primary tumor was located and a ^123^I‐mIBG scintigraphy with planar static views covering all body segments. A ^123^I‐mIBG single‐photon emission computed tomography (SPECT) with a low‐dose unenhanced (computerized tomography) CT of the body segment where the primary neuroblastoma was located was performed in 15 patients. Patients were treated with one of two standard induction chemotherapy protocols for HR‐NB, namely, either the rapid COJEC (cisplatin, vincristine, carboplatin, etoposide, and cyclophosphamide) or the modified N7 regimen (cyclophosphamide, doxorubicin, vincristine, cisplatin, and etoposide). This was followed by an evaluation of chemotherapy response with a repeat MRI and ^123^I‐mIBG scintigraphy posttreatment. Further treatment with the TVD (Topotecan‐Vincristine‐Doxorubicin) chemotherapy regime was instigated in patients with an insufficient metastatic response or unresectable primary tumor. All patients eventually underwent surgical resection.

In total, 40 patients were included in the study. The mean patient age was 2.7 years (range 0.5–7.2 years), and 21/40 patients were male. Tumor characteristics are presented in Table 1. Fifteen patients received ^123^I‐mIBG SPECT/CT imaging in addition to planar images, with the remaining patients receiving ^123^I‐mIBG scintigraphy with planar images only. ADC maps were available for the entire cohort. Regarding the induction chemotherapy protocol, 38/40 patients underwent a rapid COJEC regimen, while the other two patients received the modified N7 regimen. Five patients also required two courses of TVD regimen before surgery, while another patient required four courses of TVD with two doses of Irinotecan/Temozolomide.

**TABLE 1:** Tumor Characteristics Before and After Chemotherapy

	Prechemotherapy	Postchemotherapy
Tumor volume (mean ± SD)	245 ± 171 cm^3^	25 ± 28 cm^3^
^123^I‐mIBG avidity		
0	4	15
+	14	13
++	8	5
+++	14	7
ADC_25pcr_ (mean ± SD; x10^−3^ mm^2^/s)	0.70 ± 0.30	0.94 ± 0.45
Tumor histology		
Nonviable	0	5
Ganglioneuroblastoma	0	2
Neuroblastoma	40	30
Unknown	0	3
% of viable tumor at histology after surgical resection		
0	−	5
<10%	−	6
10–50%	−	16
>50%	−	9
Not specified	−	4

Values represent number of patients unless stated otherwise.

SD = standard deviation. ADC_25prc_ = 25^th^ percentile apparent diffusion coefficient.

### MRI

MRI scans were performed under general anesthesia with a 1.5T scanner (MAGNETOM Avanto, Siemens Healthcare, Erlangen, Germany) equipped with 40 mT/m gradients. Depending on patient size, one or two body matrix coils were used to obtain full coverage (six element design, Siemens). Axial DW‐MRI was acquired during free‐breathing, using a 2D single‐shot echo‐planar imaging sequence with b‐values of 0 and 1000 s/mm^2^. The DWI protocol was as follows: relaxation time / echo time (TR/TE): 2800/89 msec; field of view: 350 × 350 mm; voxel size: 1.4 × 1.4 mm; slice thickness: 6 mm; number of slices: 19. Nine averages were acquired for each b‐value, and trace images (mean over three orthogonal directions) were used for analysis. ADC maps were produced directly by the scanner. In addition, conventional imaging sequences were acquired in each patient according to our institution's clinical protocol, which included coronal and axial 2D, dual‐echo, short‐tau inversion recovery (STIR) sequences, axial T_2_‐weighted fast spin‐echo, and fat‐suppressed T_1_‐weighted images before and after intravenous administration of gadolinium‐based contrast agent (details in Ref. 8).

### *^123^I‐mIBG* *Scintigraphy*

^123^I‐mIBG scintigraphy was performed 24 hours after tracer injection under thyroid blockade with potassium perchlorate 10 mg/kg (from January 2006 to June 2011) or potassium iodate tablets (variable dose according to the patient's age, from 42.5 mg to 170 mg, from June 2011 to August 2019). ^123^I‐mIBG injected activity was scaled according to the patient's weight following the recommendations of the Administration of Radioactive Substances Advisory Committee (ARSAC) with doses ranging from 110 and 370 MBq.^123^I‐mIBG scans in young patients (up to 5 years of age) or in older uncooperative patients were performed under general anesthesia. Planar static images covering all body segments were acquired. From June 2008 to August 2019, a low‐dose CT scan for attenuation correction and anatomical colocalization was also acquired at the end of the SPECT acquisition (15/40 patients). Acquisition parameters were taken from the guidelines of the European Association of Nuclear Medicine.^10^, ^18^ The field of view of the CT was determined by the planar and SPECT images, as well as by other findings of interest demonstrated on MRI. The primary NB was always included in the CT field of view.

### Histopathology

Tissue specimens obtained after surgical resection of the primary tumor were examined in the histopathology department of our institution. Due to the small size of our cohort, tumors were grouped using the following categories: nonviable, ganglioneuroblastoma, and neuroblastoma (including differentiating, poorly differentiated, and undifferentiated subtypes). Clinical histopathological reports of the surgically excised tissues were collected, in which the percentage of viable tumor was classified using the following categories: nonviable, <10% viable, 10–50% viable, and >50% viable.

### Image Processing and Assessment

**FIGURE 1:**
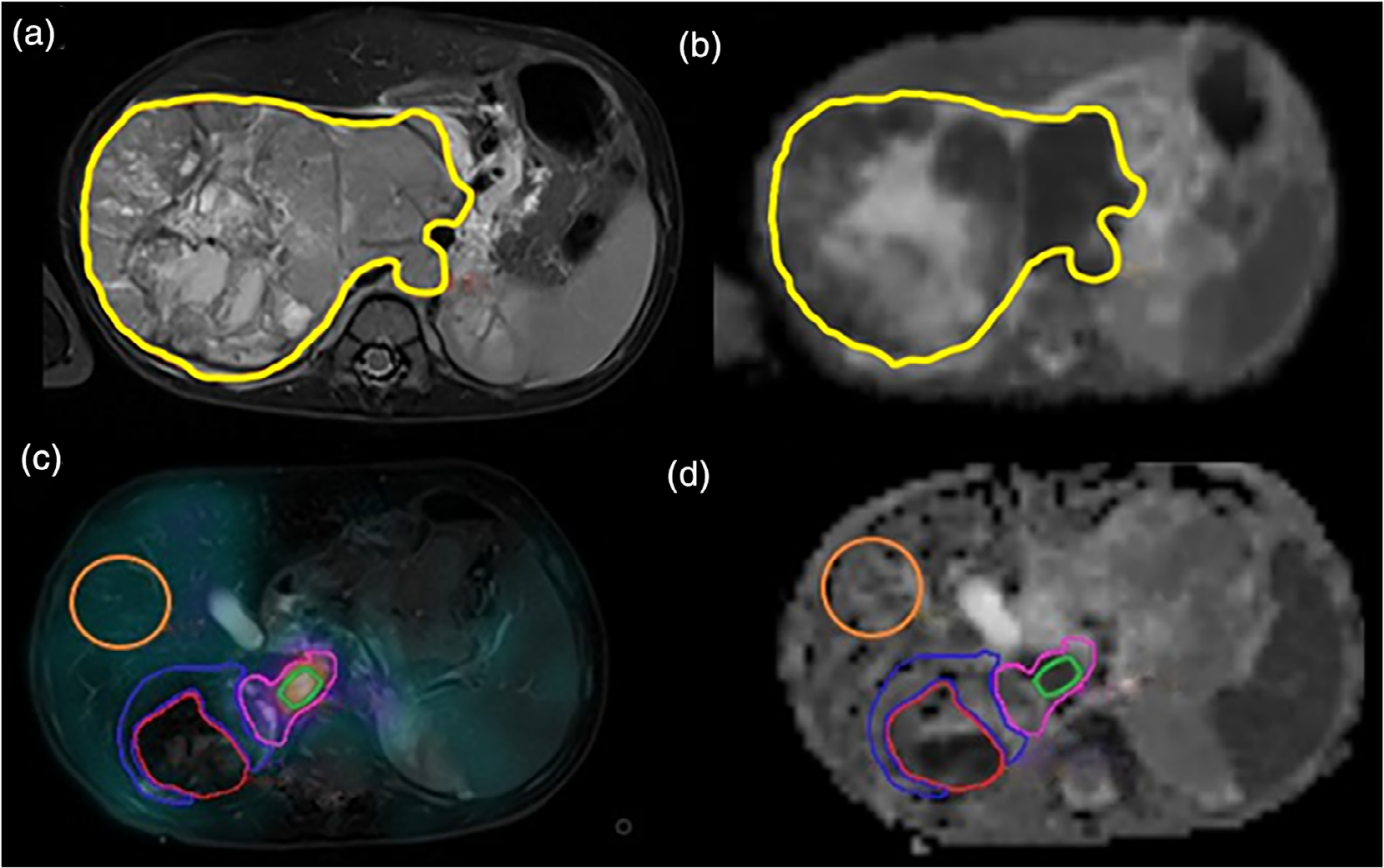
Representative images from the same patient (male, 20 months of age at diagnosis) before and after induction chemotherapy. Coregistered axial T_2_‐weighted image (**a**) and apparent diffusion coefficient (ADC) map (**b**) acquired prior to chemotherapy with the primary tumor ROI overlaid (yellow line). SPECT ^123^I‐mIBG image overlaid on T_2_‐weighted image (**c**) and ADC map (**d**) acquired postchemotherapy with the liver ROI (orange) used for normalizations and tumor subregion ROIs shown: high ^123^I‐mIBG avidity (green), moderate avidity (pink), low avidity (blue), and no avidity (red). In this example the “high avidity” ROI corresponded with the most avid part of the entire tumor volume.

Both the MRI and the ^123^I‐mIBG images were reviewed using the Hermes processing workstation (Hermes Medical Solution, Stockholm, Sweden). In every patient, an affine coregistration of the ADC maps to the T_2_‐weighted images was performed at diagnosis and after induction chemotherapy. Regions of interest (ROIs), encompassing the primary tumor volume, were drawn on each axial slice using the T_2_w images for guidance (Fig. 1a,b). ROIs delination for the study was performed by a junior surgical resident (L.P.) with 1‐year experience and a senior pediatric surgical resident (L.M.) with 5‐year experience and a special interest in neuroblastoma, closely supervised by a nuclear medicine consultant (L.B.) with 20 years of experience in pediatric nuclear medicine.

Similar to previous studies, the lower quartile ADC value (ADC_25prc_) was used to provide a representative ADC value for the entire primary tumor volume.^19^, ^20^, ^21^ The ^123^I‐mIBG uptake was assessed by the junior surgical resident (L.P.), the senior pediatric surgical resident (L.M.), and the nuclear medicine consultant (L.B.); each of them independently reviewed the planar static images and, where available, the SPECT/CT images. Consensus interpretation was obtained by both readers if there was any initial discrepant interpretation. The overall level of uptake in the primary tumor in the static planar images was graded visually as no ^123^I‐mIBG avidity (0) or mildly (+), moderately (++), or strongly (+++) avid if the uptake was lower than, similar to, or higher than the average uptake of tracer in the entire liver, respectively.

In the subcohort of patients where SPECT/CT data were available, each patient's ^123^I‐mIBG SPECT/CT study was coregistered to the T_2_w images acquired at the corresponding timepoint (either at diagnosis or at the end of chemotherapy). This coregistration was performed by the junior surgical resident (L.P.), closely supervised by the senior nuclear medicine consultant (L.B.). The axial SPECT images were coregistered to the MRI axial T_2_w images using the Hybrid Viewer software (v. 5.1) available on the nuclear medicine Hermes processing workstation: the program rigidly colocalizes the MRI and SPECT images; the coregistration was then optimized by manually adjusting the image translations using anatomical landmarks such as the normal physiological ^123^I‐mIBG liver and myocardial uptake and the ^123^I‐mIBG excretion in the bladder.

Following this, in each axial slice of the coregistered images, the whole‐tumor ROI was segmented into different subregions, based on the visual assessment of the ^123^I‐mIBG avidity in comparison to the same patient's liver ^123^I‐mIBG uptake. These were nonoverlapping regions that demonstrated strong (+++), moderate (++), mild(+), or no avidity (0) at visual assessment. In addition, a single ROI was drawn on the most avid subregion of the tumor on a single axial slice (Fig. 1c,d).

To provide a semiquantitative measure of ^123^I‐mIBG uptake, an additional 3‐cm diameter circular ROI was drawn on the right lobe of the liver in this subset of 15 patients. Raw ^123^I‐mIBG uptake values obtained from the different subregions of the tumor were compared to liver uptake estimated using the liver ROI: a tumor‐to‐liver count ratio (TLCR) was calculated by dividing the median uptake value of each subregion within the tumor by the median uptake value in the liver ROI. Median ADC and TLCR values were recorded from the tumor subregions on each axial slice.

### *Association Between* *ADC* *and* *^123^I‐mIBG* *Avidity*

The association between ADC values and ^123^I‐mIBG avidity was evaluated on both a “whole‐tumor” basis (using the full cohort of 40 patients) and an intratumor basis (using the subcohort of 15 patients where a SPECT/CT study was available). For the whole‐tumor analysis, patients were grouped based on the avidity of their primary tumor (ranging from no avidity [0] to strongly avid [+++], as described above). A one‐way analysis of variance (ANOVA) was used to examine differences in whole‐tumor ADC_25prc_ values across these groups, followed by a post‐hoc analysis using Tukey's honestly significant difference criterion to correct for multiple comparisons. This was performed independently for the pre‐ and postchemotherapy data.

For the intratumor analysis, median ADC and TLCR values were recorded from the tumor subregions on each axial slice. Using these data, provided at least three subregions were available, the intratumor correlation between ADC and TLCR was examined, using the *fitlm* function in MatLab (MathWorks, Natick, MA). This was performed independently for each patient at both timepoints (pre−/postchemotherapy). An additional linear model was used to test the correlation between median ADC and TLCR values obtained from the most avid part of each tumor. The *P*‐values associated with each linear model were corrected for multiple comparisons using the Bonferroni–Holm method.

### Treatment‐Induced Changes

As a result of chemotherapy, the association between changes in both tumor ^123^I‐mIBG avidity and ADC_25prc_ was assessed in the full cohort of patients. Using the avidity scores described above, patients were split into three groups: those in which the tumor showed a decrease in avidity as a result of treatment, those with no change, and those with an increase in avidity. A one‐way ANOVA was used to perform comparisons of the chemotherapy‐induced percentage change in whole‐tumor ADC_25prc_ values across these groups.

We also examined if pretreatment ADC_25prc_ and/or ^123^I‐mIBG avidity were predictive of a favorable response to chemotherapy. This was defined using two criteria. The first was a shrinkage in tumor size, defined as the percentage change in the volume of the entire primary tumor ROI following chemotherapy (with higher values representing greater shrinkage). The association between tumor shrinkage and the whole‐tumor ADC_25prc_ values before treatment was examined using a linear model. In addition, tumors were grouped according to their pretreatment avidity scores (0 to +++), and a one‐way ANOVA was used to examine differences in the level of tumor shrinkage across these groups.

The second criterion for assessing treatment response was the histological classification of the percentage of viable tumor remaining in the surgical specimen after treatment. The four categories described above (ranging from nonviable tumor to >50% viable) were used to group the patients, and a one‐way ANOVA was used to examine differences in pre‐ and posttreatment whole‐tumor ADC_25prc_ values across these groups. A similar analysis was performed using median whole‐tumor TLCR values; however, these quantitative values were only available for the subcohort of patients who received SPECT/CT imaging.

Finally, receiver operator characteristic (ROC) analysis was used to determine if posttreatment ADC and TLCR threshold values could be defined to stratify tumors based on their percentage of viable tumor cells. For ADC values, a threshold value was determined to separate nonviable tumors from those with some level of viable residual tumor. For TLCR values, a threshold value was determined to separate tumors with 10–50% viable tissue from those with >50% viable tissue, as all tumors in the subcohort with SPECT/CT imaging contained >10% viable tissue following treatment.

### Stratification of Histological Subtypes

Group differences in postchemotherapy ADC_25prc_ values between the nonviable, ganglioneuroblastoma, and neuroblastoma histological subtypes were assessed using a one‐way ANOVA. For the postchemotherapy ^123^I‐mIBG avidity scores, a 4 × 3 contingency table was created, with the three columns representing the histological subtypes, and the four rows representing the avidity scores (0 to “+++”). A chi‐square test was used to assess the association between the two variables.

### Statistical Analysis

All statistical analysis was performed using MatLab (v. R2019b). All *P*‐values were corrected for multiple comparisons, using Tukey's honestly significant difference criterion and Bonferroni–Holm correction as described above, following which *P* < 0.05 was considered the threshold for significance.

## Results

### *Association Between* *ADC* *and* *^123^I‐mIBG* *Avidity*

**FIGURE 2:**
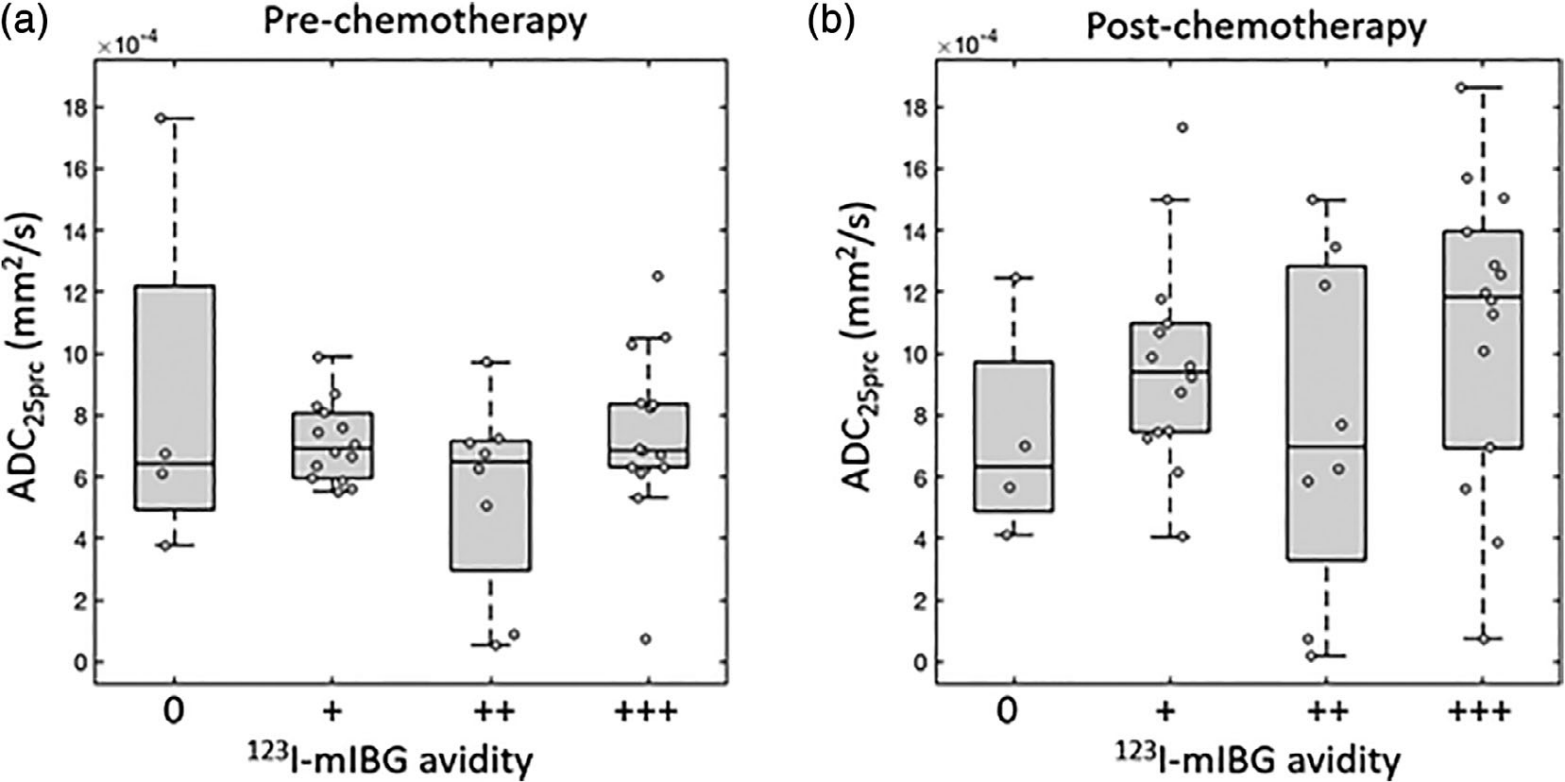
Box‐and‐whisker plots of 25^th^ percentile apparent diffusion coefficient values (ADC_25prc_) in each patient's primary tumor at prechemotherapy (**a**) and postchemotherapy (**b**) timepoints. Primary tumors are grouped according to the qualitative assessment of their ^123^I‐mIBG avidity as follows: non‐avid (0), mildly (+), moderately (++), or strongly (+++) avid. Data points for individual tumors are overlaid (circles).

The distribution of whole‐tumor ADC_25prc_ values across the ^123^I‐mIBG avidity groups is shown in Fig. 2. The difference in ADC_25prc_ values between the avidity groups was not significant at either timepoint (*P* = 0.31 prechemotherapy, *P* = 0.35 postchemotherapy).

**FIGURE 3:**
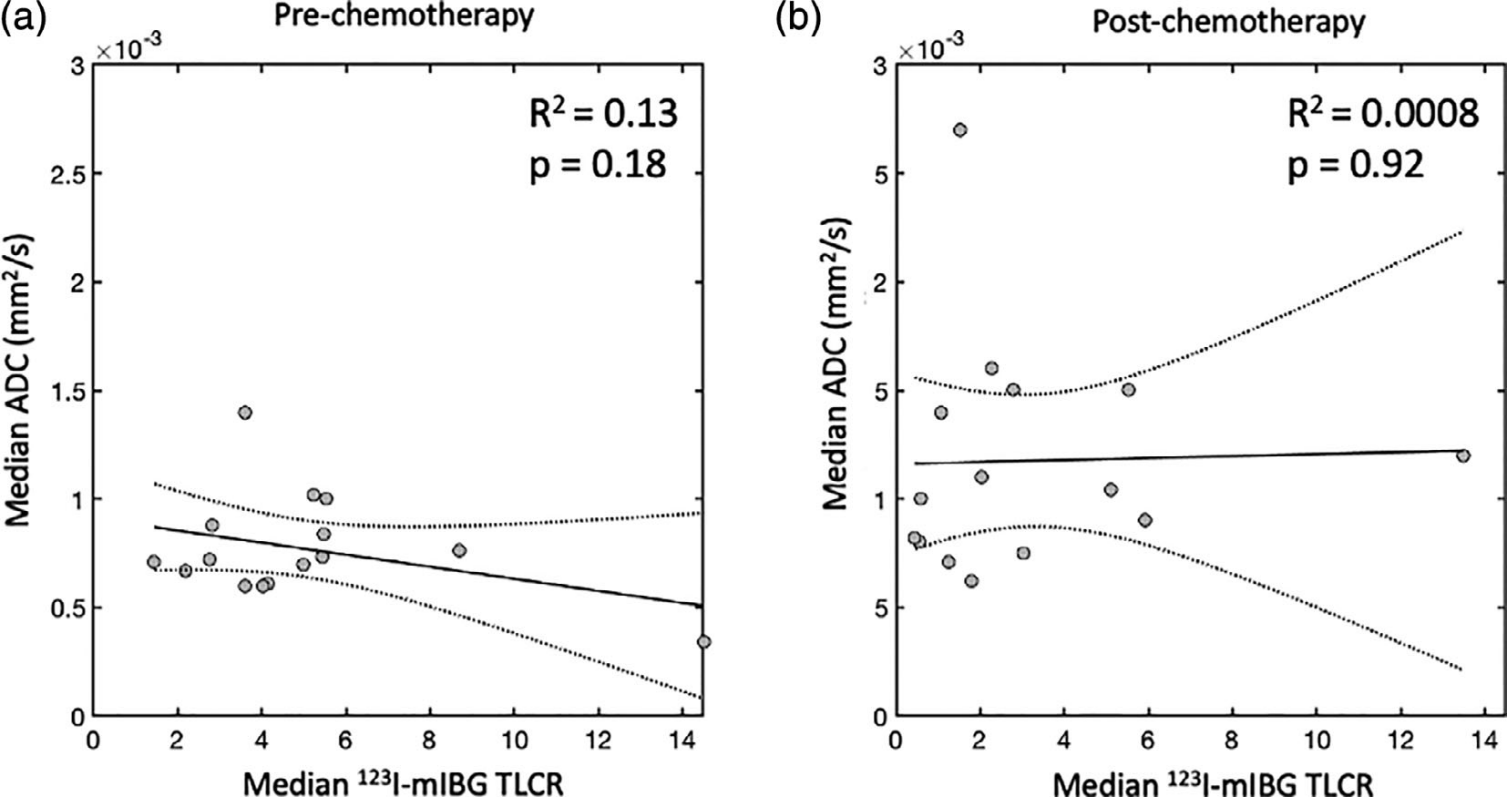
Plots of median ^123^I‐mIBG tumor‐to‐liver count ratio (TLCR) values vs. median apparent diffusion coefficient (ADC) values in the most avid part of each primary tumor. Data points (circles) represent values from each individual tumor with the trend line and associated 95% confidence interval shown as solid and dashed lines, respectively. Data are shown before (**a**) and after (**b**) chemotherapy.

The results from the individual linear models, used to test the association between median ADC and TLCR values in the subset of 15 patients who received SPECT/CT imaging, are presented in Table 2. It was possible to define at least three distinct tumor subregions across the tumor volume (using all axial slices) for all patients prior to chemotherapy (mean = 32 subregions). After chemotherapy, it was possible to do the same in 14/15 patients (mean = 9 subregions). In one patient (patient 12 in Table 2), the primary tumor shrunk considerably after treatment, and only two distinct subregions could be defined, and as such, this was excluded from the postchemotherapy intratumor analysis. The intratumor linear models revealed a significant negative correlation between ADC and TLCR in only 5/15 tumors before chemotherapy (*P* < 0.05 after multiple comparison correction, Table 2), with lower ADC values being associated with higher TLCR values. Following chemotherapy, no significant correlation was found between ADC and TLCR values in all 14 patients (Table 2). Similarly, no significant correlation was found between median ADC and TLCR values in the most avid part of each primary tumor, at both the prechemotherapy (R^2^ = 0.13, *P* = 0.18) and postchemotherapy (R^2^ = 0.0008, *P* = 0.92) timepoints (Fig. 3).

**TABLE 2:** R^2^ and *P* Values (Following Multiple Comparison Correction) for the Intratumor Linear Models Used to Explore the Association Between 25^th^ Percentile Apparent Diffusion Coefficient (ADC_25prc_) and Tumor‐to‐Liver Count Ratio (TLCR) Values in Each Patient

	Prechemotherapy		Postchemotherapy	
Patient no.	R^2^	*P* value	R^2^	*P* value
1	0.121	0.628	0.069	1.000
2	0.020	1.000	0.016	1.000
3	0.017	1.000	0.068	1.000
4	0.577	0.042*	0.925	1.000
5	0.351	0.046*	0.253	1.000
6	0.192	0.314	0.129	1.000
7	0.011	1.000	0.036	1.000
8	0.132	1.000	0.006	1.000
9	0.028	1.000	0.002	1.000
10	0.706	0.017*	0.018	1.000
11	0.053	1.000	0.053	1.000
12	0.303	0.002*	N/A	N/A
13	0.283	0.179	0.000	1.000
14	0.271	0.012*	0.004	1.000
15	0.082	0.583	0.173	1.000

*Significant correlation (*P* < 0.05). In patient 12, only two distinct tumor subregions could be identified postchemotherapy, which prevented a linear model being tested at this timepoint.

### Treatment‐Induced Changes

**FIGURE 4:**
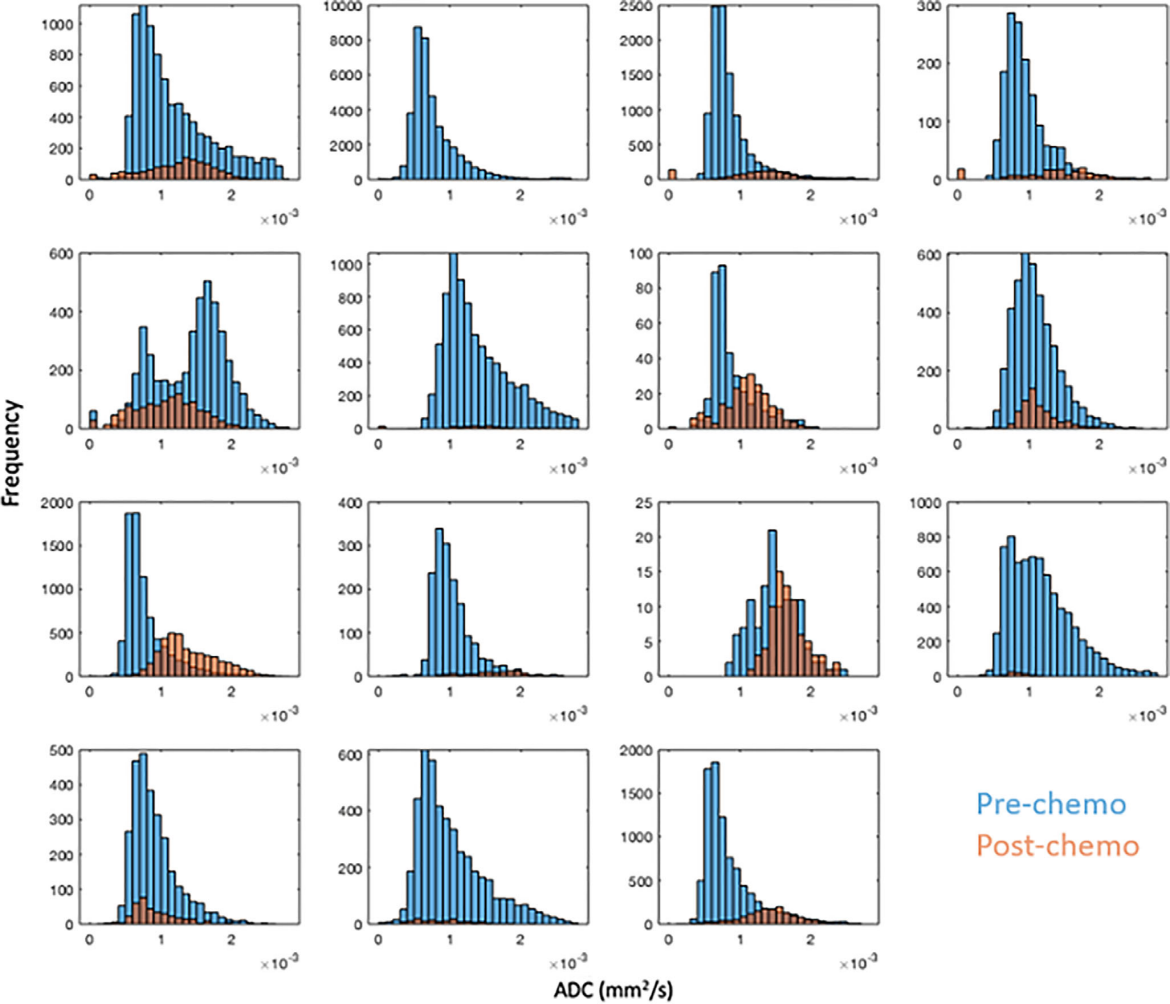
Apparent diffusion coefficient (ADC) value distributions on a whole tumor basis from the subcohort of 15 patients who receieved SPECT/CT imaging, at the prechemotherapy (blue) and postchemotherapy (orange) timepoints.

A summary of the treatment‐induced changes in our cohort is shown in Table 1. Following chemotherapy, tumor volumes shrank by an average of 90%. Before treatment, ^123^I‐mIBG avidity in the majority of tumors was scored as either “+” (14/40 patients) or “+++” (14/40 patients). Following treatment, the majority of tumors were scored as “0” (no‐avidity, 15/40 patients) or “+” (13/40 patients). The mean value of ADC_25prc_ across the cohort increased by 34% (Table 1 and Fig. 4).

**FIGURE 5:**
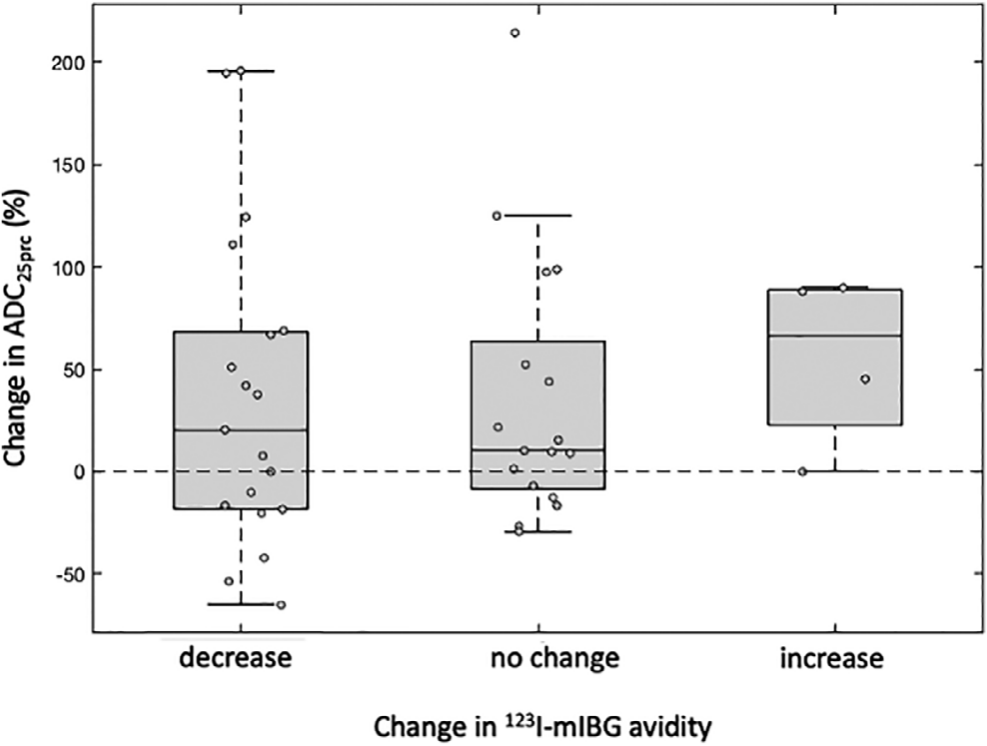
Box‐and‐whisker plots of chemotherapy‐induced percentage change in whole‐tumor 25^th^ percentile apparent diffusion coefficient (ADC_25prc_) values, grouped by change in ^123^I‐mIBG avidity in the same tumor.

**FIGURE 6:**
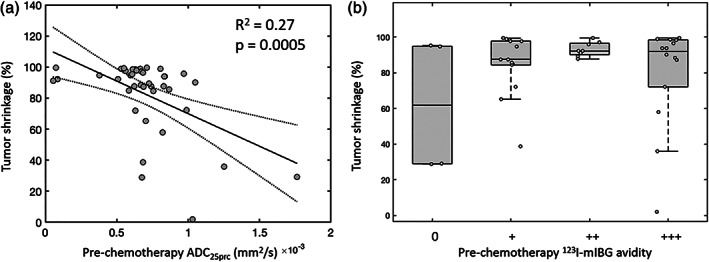
(**a**) Plot of prechemotherapy, whole‐tumor 25^th^ percentile apparent diffusion coefficient (ADC_25prc_) values vs. tumor shrinkage following chemotherapy. Data points (circles) represent individual primary tumors, with the linear fit (solid line) and associated 95% confidence intervals (dashed lines) overlaid. (**b**) Box‐and‐whisker plots of tumor shrinkage values grouped by the level of ^123^I‐mIBG avidity in the primary tumor prior to treatment.

There was no significant difference in the change in whole‐tumor ADC_25prc_ values across tumors grouped according to their change in ^123^I‐mIBG avidity following treatment (*P* = 0.86; Fig. 5). Linear regression revealed a significant negative correlation between prechemotherapy whole‐tumor ADC_25prc_ values and tumor shrinkage following chemotherapy (R^2^ = 0.27, *P* < 0.05; Fig. 6a). There was no significant difference in the amount of tumor shrinkage across the tumors when grouped according to their pretreatment ^123^I‐mIBG avidity scores (*P* = 0.17; Fig. 6b).

**FIGURE 7:**
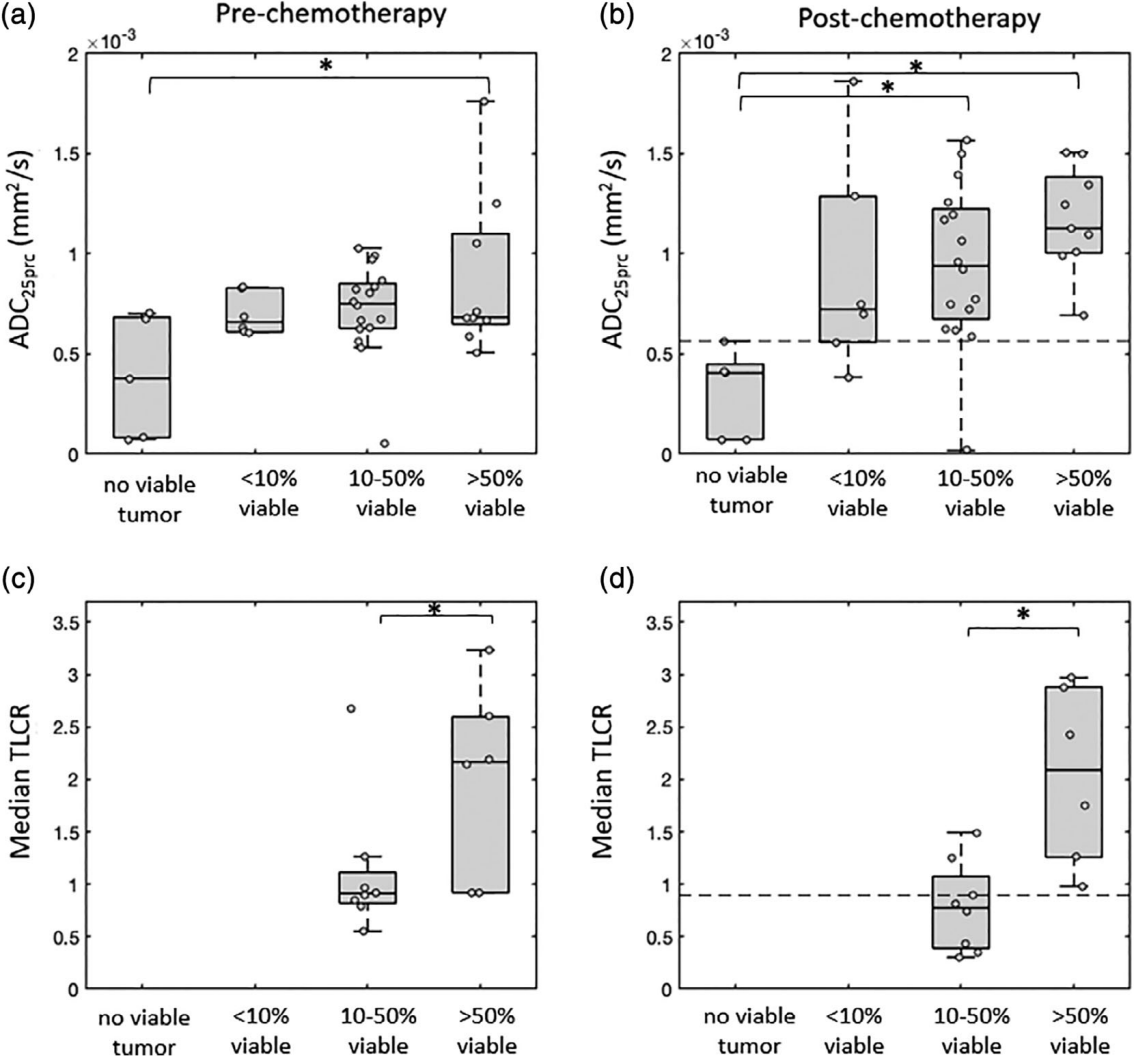
Box‐and‐whisker plots of pre‐ and postchemotherapy whole‐tumor 25^th^ percentile apparent diffusion coefficient (ADC_25prc_) values (**a,b**) and median tumor‐to‐liver count ratio (TLCR) values (**c,d**) grouped according to the level of viable tumor remaining in the excised tissue following chemotherapy. Note that fewer tumors were available for the analysis shown in (c) and (d), as quantitative TLCR values were only available in a subset of 15 patients. Significant differences between groups are indicated (*P < 0.05). The optimum threshold value for identifying completely nonviable tumors based on their postchemotherapy ADC_25prc_ values is indicated with a dashed line in (b) (ADC_25prc_ threshold = 0.57 × 10^−3^ mm^2^/s), and the optimum median TLCR threshold for identifying tumors with >50% viable tissue is indicated with a dashed line in (d) (TLCR = 0.90).

After grouping the patients according to the percentage of viable tumor cells remaining in the excised primary tumor tissue following chemotherapy, tumors with no viable tumor cells had significantly lower pretreatment ADC_25prc_ values compared to those with >50% viable tumor cells remaining (*P* < 0.05; Fig. 7a). A similar pattern was seen on posttreatment imaging, with completely nonviable tumors having significantly lower ADC_25prc_ values than both the 10–50% viable tumors (*P* < 0.05) and > 50% viable tumors (*P* < 0.05; Fig. 7b and Fig. 9 in the Supplementary Material).

Both pre‐ and posttreatment median TLCR values were significantly higher in the patients who had >50% viable tumor compared to those with 10–50% viable tumor (*P* < 0.05; Fig. 7c,d). None of the patients in the SPECT/CT subgroup had <10% viable tumor remaining following chemotherapy.

The optimum ADC threshold for separating completely nonviable tumors from those with 10–100% viable tumor on postchemotherapy imaging was ADC_25prc_ = 0.57 × 10^−3^ mm^2^/s (dashed line Fig. 7b). Tumors with a whole‐tumor ADC_25prc_ value less than this were correctly classified as completely nonviable, with 100% sensitivity and 90% specificity. In terms of median TLCR values, the optimum threshold for separating >50% viable tumors from 10–50% viable tumors was 0.90, with tumors with a median TLCR value above this being correctly classified as >50% viable with 100% sensitivity and 75% specificity (dashed line Fig. 7d).

### Stratification of Histological Subtypes

Posttreatment histology data on the surgical specimens were available for 37/40 patients and revealed the following subtypes (patient numbers in parentheses): neuroblastoma (30); nonviable tumor (5); ganglioneuroblastoma (2).

**FIGURE 8:**
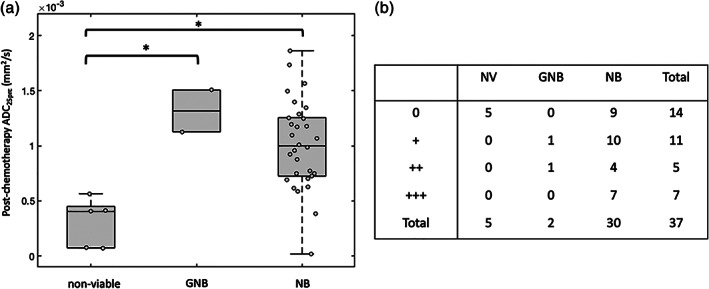
(**a**) Box‐and‐whisker plots of postchemotherapy whole‐tumor 25^th^ percentile apparent diffusion coefficient (ADC_25prc_) values, grouped by histological subtype (GNB: ganglioneuroblastoma; NB: neuroblastoma). Significant differences between groups are indicated (*P < 0.05). (**b**) 4 × 3 contingency table for postchemotherapy ^123^I‐mIBG avidity grouped by histological subtype. Numbers represent the number of patients in each cell.

Postchemotherapy whole‐tumor ADC_25prc_ values were significantly lower in nonviable tumors compared to ganglioneuroblastoma (*P* < 0.05) and neuroblastoma (*P* < 0.05; Fig. 8a). The 4 × 3 contingency table for postchemotherapy ^123^I‐mIBG avidity grouped by histological subtype is shown in Fig. 8b. There was no significant association between histological subtype and ^123^I‐mIBG avidity (chi‐squared = 12.5, *P* = 0.051); however, nonviable tumors and ganglioneuroblastoma trended towards being more prevalent in the non‐avid and lower avidity groups.

## Discussion

The aim of this study was to explore the relationship between ADC values, ^123^I‐mIBG uptake, and histopathology in primary high‐risk neuroblastomas, before and after chemotherapy. By comparing planar ^123^I‐mIBG images and the ADC values, no association was found before and after chemotherapy between “whole‐tumor” ADC and ^123^I‐mIBG uptake. We used the ADC_25prc_ value to characterize “whole‐tumor” ADC because the mean/median ADC values could be artificially inflated due to edematous nonviable regions within the tumor volume. Similarly, minimum ADC values can be corrupted by necrosis or coagulation, resulting in markedly low ADC values that mimick highly cellular tumor tissue.^22^, ^23^ However, any single ADC value used to represent the whole tumor will be susceptible to the considerable heterogeneity present within a given tumor caused by edematous, hemorrhagic, necrotic, and calcified regions. Consequently, examining the relationship between ADC values and ^123^I‐mIBG uptake on a spatially‐matched, “intratumor” basis within each patient may be a better way to evaluate whether a correlation exists. For most patients in this study, the intratumor analysis confirmed the results seen in the whole‐tumor analysis, showing a lack of correlation between ADC and ^123^I‐mIBG uptake. Although a significant negative correlation between median ADC and normalized ^123^I‐mIBG values was found prior to chemotherapy in five patients, no relevant differences were identified between these and the rest of the cohort to explain this pattern. In addition, our results showed that when only the most ^123^I‐mIBG avid part of the primary tumor is considered, there is still no correlation between the level of ^123^I‐mIBG avidity and the ADC values in this subregion.

In terms of treatment‐induced changes, our results demonstrate that changes in ^123^I‐mIBG avidity in the primary tumor are independent of changes in the ADC values, suggesting that ^123^I‐mIBG uptake is not influenced by the cellular density of the tumor. This indicates that ^123^I‐mIBG avidity and the ADC values provide different information about tumor biology. As from previous reports, ^123^I‐mIBG uptake is likely to indicate the presence of viable neuroblastoma cells but may not necessarily correlate with the aggressiveness of the disease.^15^, ^24^, ^25^

In terms of predicting tumor shrinkage as a result of chemotherapy, tumors that started with lower ADC values (prechemotherapy) showed increased levels of shrinkage. We did not find a similar pattern in pretreatment ^123^I‐mIBG avidity scores; therefore, ADC may be a more useful tool for predicting chemotherapy‐induced tumor shrinkage. However, it should be noted that a reduction in tumor volume, while being advantageous from a surgical point of view, does not necessarily indicate a favorable response to treatment, as tumors that shrink in volume may still harbor predominantly malignant cells.^5^, ^26^

The potential of DW‐MRI as a noninvasive biomarker in neuroblastoma at diagnosis and after chemotherapy is so far not well understood. In a small group of patients, low baseline ADC has been shown to be predictive of tumor progression and relapse in NB patients.^16^ In this study, increasing ADC values following chemotherapy appeared to predict relapse‐free survival, while a decreasing ADC was an indicator of poor prognosis. Another study aimed to evaluate whether ADC values in viable portions of childhood neuroblastomas showed any association with tumor cellularity: no significant correlation was found between the ADC values of the primary tumors after chemotherapy and their cellularity after resection.^27^ Our results indicate that neuroblastomas histologically classified as completely nonviable after chemotherapy had significantly lower pre‐ and posttreatment ADC values compared to neuroblastomas with viable residual tumor cells.

Also, in these completely nonviable tumors ADC values did not change considerably as a result of chemotherapy, which may indicate that these tumors contained a nonviable component prior to treatment. Overall, it appeared that low ADC values may be useful for identifying completely nonviable tumors. This is not concordant with the pattern seen in other solid tumors, where low ADC values are usually indicative of increased cellular density and more malignant tissue.^28^ It is likely that, in the case of neuroblastoma, hemorrhagic, necrotic, and calcified regions within the tumor volume are a major source of reduced ADC values, rather than an increased cellular density due to malignant cancer cells.

Although fewer quantitative data were available for ^123^I‐mIBG avidity, our results showed that a higher pre‐ and posttreatment avidity was observed in tumors in which >50% of the tumor cells remained viable following treatment, compared to those where 10–50% of the tumor remained viable. Hence, persistent ^123^I‐mIBG avidity may be a good indicator of residual viable tumor tissue, and possibly of resistance to chemotherapy, and tumors with a high median TLCR value (>0.90) are likely to retain a large proportion of viable tumor cells after chemotherapy. Similar results were obtained by Hishiki et al, who showed that ^123^I‐mIBG uptake in the primary neuroblastoma and postchemotherapy necrosis appeared to be in an inverse relationship, with more prominent residual uptake in less necrotic tumors.^25^

In terms of histological subtypes, our preliminary analysis did not show any significant difference in either ADC_25prc_ or ^123^I‐mIBG avidity in tumors that differentiated into the ganglioneuroblastoma phenotype, compared to the neuroblastoma phenotype. With regard to the ^123^I‐mIBG avidity, previous studies have produced discordant results.^15^, ^24^, ^25^ In a study of 23 patients, poorly differentiated neuroblastomas showed more intense uptake than differentiated subtypes.^15^ Other studies, in agreement with our results, found no significant difference in ^123^I‐mIBG uptake among histological subtypes. Overall, it appears ^123^I‐mIBG uptake is related to the amount of viable NB cells more than the degree of differentiation.^24^, ^25^

## Limitations

The total number of cases included in our study is unlikely to be sufficient to comprehensively explore the differences between histological subtypes and ^123^I‐mIBG and ADC values. Hence, a larger cohort is needed to confirm these findings. The ^123^I‐mIBG liver uptake was used as a reference value to normalize the uptake of the other subregions of the tumor, as the available software at the time of the scans did not allow obtaining a semiquantitative standardized uptake value (SUV), and this method is suboptimal in comparison to SUV quantification. Moreover, the coregistration of ^123^I‐mIBG and MRI images was challenging, due to poor anatomical detail in the SPECT/CT images, and the fact that the two studies were performed on different days with the patient possibly in slightly different positions. Therefore, slight inaccuracies in the coregistration could not be excluded. In addition, the coregistration of the DWI and T_2_w images is likely to contain some inaccuracies, due to the different acquisition protocols used for each modality. In addition, although our ADC_25prc_ threshold appears to be effective in identifying completely nonviable tumors, only five tumors were histologically classified as completely nonviable, and as such these results need to be further validated.

## Conclusion

The results of our study suggest that ^123^I‐mIBG avidity and ADC values do not correlate in neuroblastoma before and after chemotherapy. Instead, they characterize different aspects of tumor biology, and both have potential as noninvasive biomarkers of neuroblastoma response to chemotherapy.

## Conflict of Interest

Lorenzo Biassoni: honorarium from Siemens for an invited lecture. Elizabeth Morris: recipient of a part‐time National Institute of Health Research (NIHR) Doctoral Fellowship (NIHR300203); the work presented in this article does not form part of her fellowship. No conflict of interest declared by the other authors.

## Supporting information


**Appendix S1.** Supporting information.
