# Supplementary material for: Comparison Between Diffusion‐Weighted MRI and 123I‐mIBG Uptake in Primary High‐Risk Neuroblastoma
Source: J Magn Reson Imaging. 2020 Dec 6;53(5):1486–97. doi: 10.1002/jmri.27458 (PMC8246892; doi:10.1002/jmri.27458)
Supplement: Supplementary file 1 — Appendix S1. Supporting information. [file JMRI-53-1486-s001.docx]

**Figure 9**


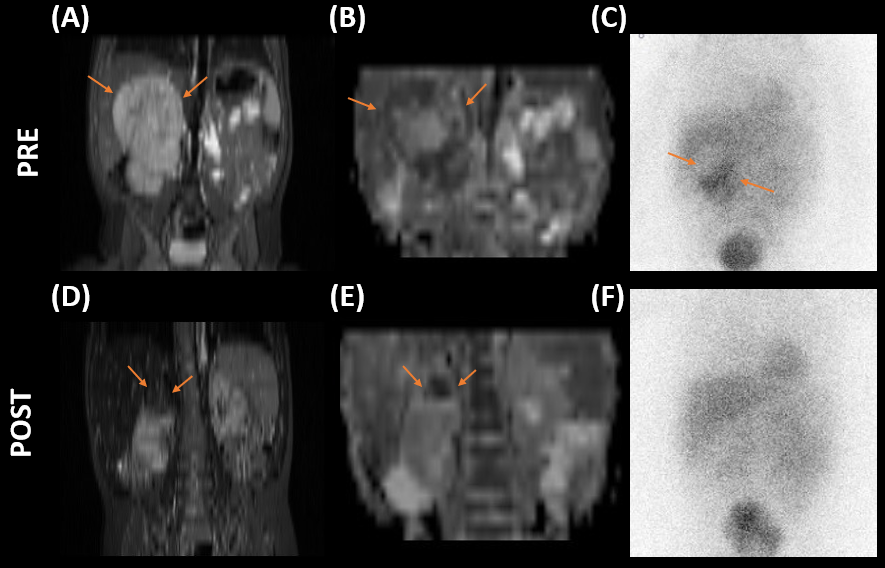


Representative images from the same patient (male, 25 months of age at diagnosis) before and after induction chemotherapy. The orange arrows indicate the neuroblastic mass. Pre-chemotherapy: the T2-weighted sequence (A) shows a right supra-renal mass lesion with heterogeneous enhancement; on the ADC map (B) the tumor shows patchy restricted diffusion with a central core of less restricted diffusion; the ^123^I-mIBG scintigraphy (C) shows a peripheral rim of strongly increased uptake in the mass lesion with a photopenic central area. Post-chemotherapy: on T2-weighted imaging (D) the primary neuroblastoma has significantly decreased in size and continues to show restricted diffusion on the ADC map (E); the ^123^I-mIBG scintigraphy (F) shows no residual ^123^I-mIBG avidity in the primary neuroblastoma. The tumor was histologically classified as completely non-viable after chemotherapy. T2-weighted and ADC maps were acquired axially and re-sliced in coronal views here to aid comparison with ^123^I-mIBG scintigraphy.
